# Automatic for the kids: assessment of cognitive efficiency in elementary school children’s auditory semantic processing

**DOI:** 10.3389/fpsyg.2025.1664852

**Published:** 2025-12-19

**Authors:** Patrick Dahdah, Igor Osipov, Johannes Naumann

**Affiliations:** Institute of Educational Research, University of Wuppertal, Wuppertal, Germany

**Keywords:** time on task, listening comprehension, cognitive efficiency, semantic processing, automaticity

## Abstract

The present study examined time on task effects in semantic processes of listening comprehension on the word and sentence levels. The aim was to investigate whether response times in auditory semantic tasks show systematic relations with accuracy, and whether these tasks therefore provide valid information about the efficiency of semantic listening processes. A total of 480 German elementary school children in Grades 1–4 solved either a semantic categorization task (*n* = 205) or a sentence verification task (*n* = 275). Accuracy and response time data were analyzed using generalized linear mixed models. Random effects for persons and items were added to test whether the time on task effects were moderated by individual skill and item difficulty. Results showed that faster responses were associated with higher accuracy for both tasks. Moreover, this relationship was stronger for more skilled comprehenders and easier items. These findings suggest that response times add meaningful information about the efficiency of semantic listening processes and may complement accuracy-based measures in the assessment of listening component skills.

## 1 Introduction

Listening comprehension plays a central role in the language development of children and serves as a foundational skill for literacy acquisition (Kim and Pilcher, 2016). Although listening comprehension develops naturally and – at least for first language acquisition – without the need for formal instruction, listening is an active and multifaceted process in which listeners attend to an auditory source, understand vocabulary and grammar, interpret paraverbal information, and retain and integrate this within a larger context (Vandergrift, 1999). Therefore, listening is directly affected by language variables such as vocabulary, grammatical knowledge, and inference skills, and has been shown to draw on cognitive resources such as working memory and attentional control, which are necessary for maintaining and integrating incoming information into a coherent mental model (Florit et al., 2009; Kim, 2015a, 2016; Lervåg et al., 2018). According to Imhof’s (2010) model of listening, which is based on the Select-Organize-Integrate (SOI) model (Mayer, 1996), listeners first select relevant auditory and visual input for further processing. This incoming information must then be organized into meaningful units by recognizing words and accessing the mental lexicon, and by determining the grammatical structure and relationships between elements. Finally, the input is integrated with prior knowledge into a coherent model, which in turn helps to monitor the comprehension process and make necessary adjustments (Imhof, 2010). As auditory information is transient, working memory plays an important role in retaining the input long enough to organize it and build a coherent text representation. Especially when lower-level language processes, such as lexical access, are not yet efficient, listeners rely more heavily on contextual information to integrate meaning on the sentence or text level (Nation and Snowling, 1998; Stanovich et al., 1985). This increased reliance on context puts a higher burden on cognitive resources and leaves fewer resources for higher-order processes, such as drawing inferences or monitoring comprehension (Imhof, 2010; Perfetti, 1985, 1992).

The limitations of cognitive resources and the related consequences of inefficient language retrieval processes make it necessary that these processes become automatic for successful comprehension (Just and Carpenter, 1992; Just et al., 1996; Perfetti, 1985). We use the term *automatic* to represent rapid and low-effort access to mental representations under task-directed attention, in line with the verbal efficiency theory (Perfetti, 1985). We further use the term cognitive efficiency to describe the same phenomenon. Hui and Godfroid (2021) found that, for second language listeners, automaticity especially in lexical processes was important for propositional comprehension and in turn for general listening comprehension. This is analogous to processes in reading comprehension, in which the efficiency of word recognition and automatic activation of semantic networks support the efficiency in reading comprehension (Kim, 2015b; Perfetti, 1985, 1992). For listening, not only the transiency, but also the lack of control regarding the spoken input (i.e., the speed or amount of incoming information) can further lead to impaired comprehension when at least lexical-level comprehension processes are not automatic (Hui and Godfroid, 2021). Thus, whereas attentional resources play an important role especially for higher-level listening processes (e.g., Kim, 2015a; Shtyrov, 2010), automaticity in the processing of incoming oral language information and in the activation of semantic networks plays an important role in the efficiency and success of listening comprehension.

These considerations have implications for the assessment of listening skills. If automatic processes play a key role in listening comprehension, assessment procedures should not only measure how accurately listeners understand spoken language, but also how cognitively efficient they do so – that is, the extent to which listeners process spoken language quickly and with minimal cognitive effort. In the related cognitive domain of reading, a couple of processes, such as decoding or phonological recoding, but also sentence level processes such as semantic integration on the sentence level, are amenable to become automatic, and are automatic in experienced readers. Consequently, a number of reading tests that assess word- or sentence level processes have a speed component such that test scores increase not only with more correct responses, but also with shorter response times. This applies to paper-based tests at the sentence level that require judging as many sentences as possible within a given time (e.g., *Salzburger Lese-Screening* [*Salzburg reading screening*], SLS 2–9; Wimmer and Mayringer, 2014), to paper-based, word-level tests that require reading and understanding words quickly (e.g., *Würzburger Leise Leseprobe* [*Würzburg silent reading probe*], WLLP-R, Schneider et al., 2011), and to computer-based test batteries of reading component skills where item-level response times are integrated with accuracy to estimate proficiency (e.g., *Prozessbezogene Diagnostik von Lesefähigkeiten im Grundschulalter* [*Process-oriented measurement of reading skills in primary school children*], ProDi-L, Richter et al., 2017). In all these assessment procedures, the underlying assumption is that shorter response times are indicative of more highly automatized cognitive processes. As automatic cognitive processing is accurate and fast at the same time, given the right input (Shiffrin and Schneider, 1977), shorter response times are interpreted as indicative of higher proficiency levels.

The idea that short response times are akin to cognitive processes being highly routinized and thus fast and accurate at the same time is not without alternative and does not necessarily apply across the board. Specifically, in domains where tasks need a high degree of deliberate and controlled cognitive processing, such as problem solving (e.g., Nicolay et al., 2022), or hypertext reading (e.g., DeStefano and LeFevre, 2007), different interpretations of response times might apply. In these kinds of tasks, short response times might actually indicate disengagement from the task rather than automaticity or efficiency of cognitive processes. Where a task requires controlled cognitive processing to a large degree, and thus the investment of time, walking away from it by giving a quick response already might be a recipe for failure. Moreover, also within domains, time on task might have different associations with the odds of success, depending on both tasks and persons. In the domain of reading, for example, Walczyk (2000) proposed a model (“compensatory encoding model”) that claims that especially weak readers might compensate for their lack of skills by investment of additional time on task. From this perspective, when a task is cognitively challenging, response time and accuracy might be positively, rather than negatively related, and more so the less able a person is within the respective domain and the more difficult a task is.

A number of studies, both across and within domains, have provided evidence that indeed time on task effects are a function of tasks involving predominantly cognitive processes that are deliberate and consumptive of working memory, or largely automatic. First evidence for this idea was provided in a study by Goldhammer et al. (2014). These authors used field trial data from the OECD Programme for the International Assessment of Adult Literacies (PIAAC; see OECD, 2012) to investigate the relation between time on task and accuracy in two domains, problem solving in technology-rich environments and reading. They argued that problem solving is an activity that draws heavily on controlled cognitive processing, which follows almost from the definition of a “problem”: A problem exists if there is a discrepancy between a given and a goal state that, other than in a routine task, cannot be overcome by routine operations (Frensch and Funke, 1995). In such a situation, deliberate and controlled cognitive processing will be required to devise means suited to bridge the gap between the given and the goal state (Mayer and Wittrock, 2006).

In contrast to this, the reading tasks employed involved only the reading of single linear texts, thus involving cognitive operations from word decoding to the formation of a situation model, which most adult readers can carry out by means of automatic processing to a large degree (Schindler and Richter, 2018). In line with the authors’ hypotheses, in a generalized linear mixed model, the mean (fixed) effect of time on task was positive for problem solving, and negative for reading. Moreover, within domains, time on task effects were moderated by persons’ skill and items’ difficulty, as shown by the random effects’ covariance structure, that allowed random intercepts for persons and items to co-vary with random time on task slopes across persons and items. Positive time on task effects in problem solving increased with decreasing skill and increasing difficulty. Likewise, negative time on task effects in reading increased with increasing skill and decreasing difficulty. Thus, in problem solving, investment of time was crucial especially in hard tasks, and paid off especially for weaker problem solvers. In contrast, in reading, the association between speed and accuracy was strongest for easier tasks and skilled readers. This pattern of results was interpreted by the authors as being consistent with a dual-processing account (see Schneider and Shiffrin, 1977; Shiffrin and Schneider, 1977) of reading and problem solving, where reading tasks as those that were used in the PIAAC assessment are reliant on cognitive processes that are largely amenable to automatization, while problem solving requires controlled cognitive processing to a large degree. Further research extended and corroborated the idea that time on task is positively predictive of performance in complex cognitive tasks that require deliberate and controlled processing (Naumann and Goldhammer, 2017; Scherer et al., 2015; Vörös et al., 2021).

Vörös et al. (2021) replicated and extended the problem-solving part of Goldhammer et al.’s (2014) study and analyzed PIAAC problem solving data that came from the main assessment rather than the field trial. Drawing on this stronger data base, they found the same result that time on task effects were positive, and even more so in harder items. Moreover, they showed that task characteristics such as text complexity or technology load further increased the overall positive time on task effect, supporting to the idea that in problem solving, mobilization of cognitive resources requires taking time, and thereby positively contributes to task solution. Scherer et al. (2015) analyzed data from 2000 Finnish youths who completed an assessment of Complex Problem Solving. Using a structural equation modeling approach rather than a generalized mixed model as in Goldhammer et al. (2014), the authors also found positive associations between time on task and performance in solving complex problems. Moreover, both time on task and performance were positively predicted by reasoning ability, meaning that more intelligent students both performed better and spent more time to complete the assessment’s problem-solving tasks. Once again, this is in line with the idea that longer response times in complex cognitive tasks indicate not inefficiency, but engagement that adapts to the task’s difficulty. Naumann and Goldhammer (2017) extended the work by Goldhammer et al. (2014) through investigating a domain that can be viewed as comprising elements of both reading and problem solving, namely digital or hypertext reading, thus reading in an environment where it is left to the reader to select and integrate information from multiple sources according to both the sources’ credibility and usefulness. Such reading situations have explicitly been modeled as *Reading as Problem Solving* (RESOLV, Rouet et al., 2017) and have been shown to rely on problem solving skills (Naumann et al., 2025). From this perspective, the authors expected time on task effects that more closely resemble those observed in problem solving than those found for reading linear paragraphs, a prediction that was confirmed: Time on task effects were overall positive, and especially so for less skilled individuals and more difficult tasks. Moreover, they found that the features of digital text that require controlled processing, specifically navigation demands, moderated time on task effects. These effects were especially positive for items with high navigation demands.

In contrast to tasks that require controlled cognitive processing, reading tasks targeting lower-level reading processes such as phonological recoding (Naumann et al., 2016) or orthographic comparison (Naumann, 2018), tend to show negative, rather than positive, time on task effects. Notably, the magnitude of these negative time on task effects in both domains increased with increasing skill in the respective domain. In other words, especially in persons who are skilled in phonological recoding and in orthographic comparison, fast responses were also accurate. Furthermore, in the orthographic comparison domain, Naumann (2018) found that time on task effects were moderated by task difficulty, similar to the results reported by Goldhammer et al. (2014) for linear paragraphs: Negative time on task effects were strongest in easier tasks, likely because the item stimuli in these tasks were especially suited to be answered utilizing automatic cognitive processing. These results are in line with the view that word- and sentence-level reading component processes rely heavily on automatic processing, and that individual differences in word-level reading skill arise from differential automization and efficiency of these processes across readers (Karageorgos et al., 2020; Schindler and Richter, 2018).

Although the relationship between processing speed and accuracy has been extensively studied in reading, it remains an open question whether comparable modeling approaches can reliably capture efficiency in listening. Whereas reading and listening share many higher-order processes, especially the early, pre-comprehension processes are assumed to differ between the two which can lead to different demands on cognitive resources (Aryadoust, 2017; Imhof, 2010). In contrast to reading, listening unfolds in real time, relies on transient auditory input, and provides no opportunity for reinspection or self-paced control (Field, 2009). Listening is therefore assumed to rely more heavily on working memory and attentional resources compared to reading, where stable orthographic representations can support slower processing (Field, 2009; Florit et al., 2009; Jiang and Farquharson, 2018).

Due to this transient nature of spoken language and the resulting demands on working memory resources, listening component processes are crucial for learning and skill acquisition. From a time on task perspective, our overarching aim was to investigate whether the efficiency of listening component processes can be meaningfully assessed with tasks tapping into semantic processing, and using response times as a measure of cognitive efficiency. We thus examine whether response times systematically predict accuracy at the item and person levels, thereby testing the notion that integration of accuracy and response times could provide a more comprehensive account of efficiency in listening processes. Listening comprehension involves multiple levels of semantic processing that contribute to the construction of meaning. On the word level, listeners must efficiently access the meaning of words from their mental lexicon. On the sentence level, individual word meanings must be integrated into coherent propositions and monitored for plausibility within the sentence context. Both processes are important for successful comprehension and have been shown to rely on efficient, largely automatized processing (Imhof, 2010; Kintsch, 2005; Perfetti, 1985). In the present study, we therefore implemented tasks that measure auditory semantic processing on the word level (retrieval of word meanings) and on the sentence level (semantic integration). We investigated whether time on task effects in these tasks reflect cognitive efficiency rather than a measure of deliberate cognitive processing. To this end, we employed the parametrization introduced by Goldhammer et al. (2014) to accuracy and response time data from a semantic categorization task assessing the retrieval of word meanings, and a sentence verification task assessing semantic integration on the sentence level.

From the previously found patterns of time on task effects, and to test whether response times in these tasks show similar patterns to those associated with efficient, automatized, rather than to those of controlled or deliberate processing, we derived the following hypotheses:

*Hypothesis 1*: There is a negative time on task effect in the retrieval of word meanings, such that overall quicker retrieval of word meanings is associated with stronger performance.

*Hypothesis 2*: There is a negative association between person skill and person-specific time on task effects, such that while being negative overall, time on task effects are negative especially for persons with high levels of skill in the retrieval of word meanings.

*Hypothesis 3*: There is a negative association between item easiness and item-specific time on task-effects, such that while being negative overall, time on task effects are negative especially for easy items assessing the retrieval of word meanings.

*Hypothesis 4*: There is a negative time on task effect in the semantic integration at the sentence level so that overall quicker sentence processing is associated with stronger performance.

*Hypothesis 5*: There is a negative association between person skill and person-specific time on task effects, such that while being negative overall, time on task effects are negative especially for persons with high levels of skill of semantic integration at the sentence level.

*Hypothesis 6*: There is a negative association between item easiness and item-specific time on task-effects, such that while being negative overall, time on task effects are negative especially for easy items assessing the semantic integration at the sentence level.

## 2 Materials and methods

### 2.1 Subjects

A total of 480 elementary school children participated in the study. Of these, 205 children completed a semantic categorization task assessing the retrieval of word meanings, and 275 completed a sentence verification task assessing the semantic integration on the sentence level (see section *measures* below). Overall, 210 children were male, and 238 were female (missing gender information for 32 children). Table 1 gives the number of participating children broken down by task, gender, and grade level. From the children participating in the sentence verification task, 99 spoke German as their first language, and 50 spoke a language other than German as their first language (missing language background information for 97 children). For all children, a parent or guardian had given written consent for the children’s participation in the study. Also, school principals and teachers had given their consent for the study to be conducted in the respective schools and classrooms. For the children participating in the semantic categorization task, no information on language background was available due to schools’ data protection and privacy concerns.

**Table 1:** Number of participants per task, gender, grade level and language background.

Grade level	Semantic categorization				Sentence verification							
	Male	Female	Miss.	Total	Male			Female			Miss.	Total
					German	Other	Miss.	German	Other	Miss.		
1	14	20	0	34	0	0	9	0	0	7	22	38
2	23	36	0	59	4	5	17	3	3	14	1	47
3	25	36	1	62	46	25	10	41	29	7	1	159
4	22	22	6	50	8	1	0	16	3	0	3	31
Total	84	114	7	205	49	22	53	50	28	44	29	275

Miss., Information missing. Due to Schools’ data protection policy, no language background information is available for children participating in the Semantic Categorization task.

### 2.2 Measures

#### 2.2.1 Retrieval of word meanings

The retrieval of word meanings was measured with a semantic categorization task from the ProDi-H (*Prozessbezogene Diagnostik von Hörfähigkeiten im Grundschulalter* [*Process-oriented measurement of listening skills in primary school children*]), a test battery for the measurement of listening component skills and the auditory equivalent of the ProDi-L (Richter et al., 2017). In the task, children first heard a category word (e.g., tools), followed by a target word (e.g., hammer) 200 ms later. Of the 35 presented items, 17 featured matching category-word pairs (e.g., food-chocolate) and 18 featured non-matching pairs (e.g., plants-bed). Classification tasks like these rely on participants to retrieve word meanings from their mental lexicon (Thompson-Schill and Gabrieli, 1999) and are strongly associated with semantic processing (Kutas and Iragui, 1998; Taikh et al., 2015). Common, everyday category names were chosen to ensure that a correct response was possible when the target word was known to the children. Item difficulty varied based on word frequency, familiarity, and the degree of relatedness between category and target words. Familiarity with the categories was rated by three independent teachers on a three-level scale (unadjusted intraclass correlation ICC = 0.84; cf. McGraw and Wong, 1996). The degree of relatedness for non-matching pairs as well as the degree of typicality for the respective category for matching pairs was quantified using cosine value from Latent Semantic Analysis (LSA). The cosine values were derived from a large German semantic space (dewak100; see Günther et al., 2015). The task was computer-based and both response accuracy as well as response time were recorded.

#### 2.2.2 Semantic integration on the sentence level

Semantic integration processes were measured with a sentence verification task from the ProDi-H. Children were presented with short sentences and asked to determine whether they were true or false. This task required them to comprehend the sentence meaning and evaluate each statement based on their existing world knowledge (Richter et al., 2009). Task difficulty was influenced by two factors: (a) semantic complexity, defined by the number of propositions (which varied between 1 and 3), and (b) contextual predictability within the sentence (Frisson et al., 2005). A total of 48 sentences were presented – half of which were factually correct (Strawberries are red), and half of which were incorrect (e.g., Fire is gluey). Among the correct sentences, half were contextually predictable (e.g., Bananas are crooked and yellow) and the other half were less predictable (e.g., Flowers have roots, compared to the more expected flowers have stems). All test sentences used vocabulary that was familiar to elementary school children and maintained simple syntactic structures. Sentence difficulty for incorrect sentences was expected to be higher than for correct sentences. Furthermore, within the incorrect sentences, we assumed that a higher number of propositions would lead to more difficult items. When incorrect sentences contained more than one proposition, we assumed that those with only one false proposition would be more difficult to answer than sentences where all propositions are false. As with the semantic categorization task, both response accuracy and response time were recorded.

### 2.3 Procedure

Children were tested in their classrooms on tablet computers, with their teacher present. First, they were greeted by the experimenter, and explained that they could interrupt, or terminate their participation at any time, and if something went wrong or they had any question, they could ask for help at any time by raising their hand. The experimenter then started the script that administered the semantic categorization, or sentence verification task, respectively. Children gave their responses through the “f” and “j” keys on the keyboard. The “j” key for “yes” responses was marked with a green dot, the “f” key for “no” responses was marked with a red dot. Each task was framed within a cover story featuring an extraterrestrial “Reli,” who wanted to learn the Earthlings’ language but continued to make a lot of mistakes, and asked the children for help. “Reli” explained in detail the response keys, and that children best would keep their fingers on them during the test, but pressing only to answer an item. Children were instructed to work as fast and accurately as possible. Before the actual trials children completed two practice trials where feedback was provided. When the last child had completed the task, children were thanked for their participation and told that they or their parents or guardians could contact the researchers at any time in case they had further questions as to the purpose of the research.

### 2.4 Statistical modeling

To test for time on task effects, and their moderation by item difficulty and person skill, we used the parametrization introduced by Goldhammer et al. (2014), depicted in Equation 1. We expressed the log odds of a correct response by a fixed effect of time on task, a random person intercept, a random item intercept, and a random slope of time on task across both persons and items. Since children were clustered in classrooms, we included an additional random effect for classroom. Since children potentially varied considerably in their language proficiency due to different grade levels, we included grade level as additional three fixed effects (successive contrasts comparing grades 1–2, grades 2–3, and grades 3–4). Thus, the following (full) model was estimated:


$$\begin{array}{l} \ln(\frac{p}{1-p})=\beta_{0}+b_{0c}+b_{0p}+b_{0i}+(\beta_{1}+b_{1i}+b_{1p})\\ \cdot (\text{time}\;\mathrm{on}\;\text{task})+\beta_{2}+\beta_{3}+\beta_{4} \end{array}$$ (1)

In this notation, p is the probability that an item is answered correctly. Greek letters (*β*) refer to fixed effects, and Latin letters (b) refer to random effects. β_0_ is a fixed intercept, β_1_ is the fixed effect of time on task, β_2_, β_3_, and β_4_ are the fixed effects for grade. b_0c_, b_0p_ and b_0i_ are the random classroom, person and item intercepts, respectively. The random person intercept can be interpreted as ability, the random item intercept as easiness. (Note that the parametrization is opposite to the usual parametrization in a Rasch model where the item parameter depicts difficulty). b_1p_ and b_1i_ are the random time on task slopes, respectively. In the full model, the covariance between b_0p_ and b_1p_ is estimated, as is the covariance between b_0i_ and b_1i_.

To account for the skewness of the response time distributions, we used the natural logarithm. To facilitate model identification and estimation, in addition to taking the natural log we standardized the entire response time vector, including all items and persons. This meant that if there were differences in response time variance between persons or items, these were retained.

## 3 Results

Mean accuracy and latencies per grade level are given in Table 2. An *a priori* alpha level of 5% was set for all statistical tests. Where hypotheses were directed, tests were one-tailed. All model specifications and comparisons can be found in Table 3. The fixed effects are reported for the respective final models.

**Table 2:** Mean accuracy and response time per grade level.

Grade level	Retrieval of word meanings (Semantic categorization task)				Sentence comprehension (Sentence verification task)			
	Accuracy^a^		Response time^b^		Accuracy^a^		Response time^b^	
	*M*	*SD*	*M*	*SD*	*M*	*SD*	*M*	*SD*
1	23.12	4.86	2394.23	961.27	39.92	6.06	4464.04	1094.09
2	24.43	5.08	2262.04	710.44	40.98	5.69	4019.52	794.48
3	25.84	4.56	2347.00	819.51	42.24	4.14	3790.00	574.25
4	29.20	3.40	1980.50	504.50	44.35	2.78	3392.28	480.00

There were 35 items in the semantic categorization task and 48 items in the sentence verification task.

a Number of correctly answered items.

b Milliseconds. Note that in the analysis log-transformed response times were used that were z-standardized across all persons and items.

**Table 3:** Model specification, comparisons, and fit statistics.

Task	Model	Random slopes	Model comparison	Δ*χ*^2^ (Δ*df*)	Fixed time on task effect β_1_	Variance of random time on task effect	Correlation of random effects	AIC
Retrieval of word meanings	M0p_words_	Item	–	–	−0.13	0.03	−0.44	7,255.0
	M1p_words_	Person Item	M0p_words_ versus M1p_words_	13.18^*^ (1)	−0.16	0.08 0.03	– −0.43	7,243.9
	**M2**_**words**_	**Person** **Item**	**M1p**_**words**_ **versus M2**_**words**_	7.46^**^ **(1)**	**−0.20**	**0.08** **0.03**	**−0.53** **−0.52**	**7,238.4**
	M0i_words_	Person	–		−0.18	0.08	−0.50	7,245.5
	M1i_words_	Person Item	M0i_words_ versus M1i_words_	6.96^**^ (1)	−0.18	0.08 0.03	−0.48 –	7,240.5
			M1i_words_ versus M2_words_	4.13^*^ (1)				
	M3_words_ (no fixed grade level effect)	Person Item	M2_words_ versus M3_words_	15.11^**^ (3)	−0.22	0.09 0.03	−0.59 −0.54	7,247.5
Semantic integration in sentences	M0p_sentence_	Item	–	–	−0.41	0.07	−0.43	8,453.0
	M1p_sentence_	Person Item	M0p_sentence_ versus M1p_sentence_	23.14^***^ (1)	−0.41	0.11 0.07	– −0.37	8,431.8
	**M2**_**sentence**_	**Person** **Item**	**M1p**_sentence_ **versus M2**_sentence_	7.65^**^ **(1)**	**−0.44**	**0.11** **0.07**	**−0.38** **−0.42**	**8,426.2**
	M0i_sentence_	Person	–	–	−0.41	0.10	−0.37	8,450.8
	M1i_sentence_	Person Item	M0i_sentence_ versus M1i_sentence_	23.95^***^ (1)	−0.42	0.11 0.07	−0.35 –	8,428.8
			M1i_sentence_ versus M2_sentence_	4.64^*^ (1)				
	M3_sentence_ (no fixed grade level effect)	Person Item	M2_sentence_ versus M3_sentence_	8.41^*^ (3)	−0.45	0.10 0.07	−0.40 −0.42	8,428.6

Rows in bold indicate the selected final models. Dashes indicate that a parameter was not estimated.

^*^p < 0.05. ^**^p < 0.01. ^***^p < 0.001.

### 3.1 Retrieval of word meanings

#### 3.1.1 Fixed effects

As expected, time on task had a significant negative fixed effect, *b* = −0.20 (*SE* = 0.05), *z* = −3.83, *p* < 0.001, meaning that on average faster responses were also more accurate. This result supports hypothesis 1. While in general accuracy was higher in each successive grade descriptively (see Table 2), the respective contrasts were not significant between grades 1 and 2, *b* = 0.22 (*SE* = 0.20), *z* = 1.12, *p* > 0.05, and grades 2 and 3, *b* = 0.21 (*SE* = 0.17), *z* = 1.23, *p* > 0.05, but only between grades 3 and 4, *b* = 0.53 (*SE* = 0.19), *z* = 2.82, *p* < 0.01.

#### 3.1.2 Random effects

**Figure 1:**
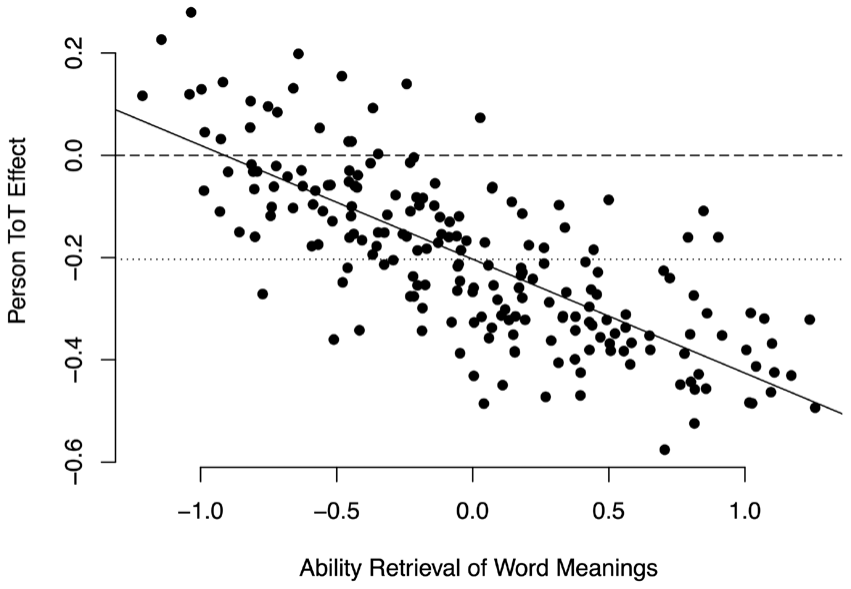
Person-specific time on task-effects in the semantic categorization task. Continuous line: Linear regression of person-specific time on task effects on ability. Dashed line: Time on task effect of zero. Dotted line: fixed time on task effect.

*Persons*. First, we examined if the time on task effect would be the same for all persons, or whether it would display variance across persons. To this end, we estimated a model that specified a random person intercept, but not a random person time on task slope (M0p_words_). This model was compared to a model that estimated a random time on task slope but constrained the covariance between intercept and slope to be zero (M1p_words_). This resulted in the model M1p_words_ specifying a random time on task slope across persons fitting the data significantly better than the model M0p_words_, *χ*^2^(1) = 13.18, *p* < 0.001. Thus, the time on task slopes varied across persons. In a next step, we examined if this variance could in part be explained by persons’ skills. We estimated a model where the covariance between the random person intercept and the random time on task slope across persons was freed (M2_words_). This resulted in a correlation of random person intercept and random time on task slope across persons of −0.53, thus a negative correlation as expected. To test this correlation for significance, we compared this model to the model M1p_words_, where the random time on task effect across persons was estimated, but could not co-vary with the random person intercept. This resulted in model M2_words_ fitting the data significantly better than model M1p_words_, *χ*^2^(1) = 7.46, *p* < 0.01. Combining the fixed time on task effect and the a posteriori estimates of the person-specific random time on task effects revealed that indeed most person-specific time on task effects were negative, and the more so the more skilled the persons were (see Figure 1). Thus, the more skilled persons were, the more fast responses were accurate at the same time. These results support hypothesis 2.

**Figure 2:**
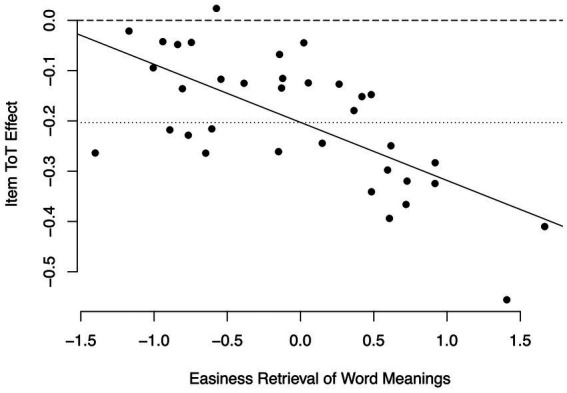
Item-specific time on task-effects in the semantic categorization task. Continuous line: Linear regression of item-specific time on task effects on item easiness. Dashed line: Time on task effect of zero. Dotted line: fixed time on task effect.

*Items*. In a first step, we examined if the fixed time on task would be the same for all items, or whether it would randomly vary across items. Thus, we first estimated a model M0i_words_ that specified only a random item intercept, but no random time on task slope across items, thus assuming that the time on task effect would be the same for all items. We then compared this model to a model M1i_words_ that specified a random time on task slope across items, but no covariance between the random item intercept and the random time on task slope across items. This resulted in the Model M1i_words_ fitting the data significantly better than the model M0i_words_, *χ*^2^(1) = 6.96, *p* < 0.01, thus time on task slopes varied across items. In a next step we examined whether this variance could be in part explained by items’ difficulties. In model M2_words_, the covariance between the random item intercept and the random time on task slope across items was freed. This resulted in a correlation of −0.52 between the random item intercept and the random time on task slope across items, thus negative as expected. To test this correlation for significance, we compared the fit of model M2_words_ to the fit of model M1i_words_, which restricted the covariance between the random person intercept and the random time on task slope across items at zero. This resulted in model M2_words_ fitting the data significantly better than model M1i_words_, *χ*^2^(1) = 4.13, *p* < 0.05. Combining the fixed time on task effect and the a posteriori estimates of the item-specific random time on task effects meant that item-specific time on task effects were negative with one exception, but it was especially in easy items that fast responses were associated with correct responses (see Figure 2). In (relatively) difficult items, the item-specific time on task effects were negative as well, but to a smaller extent. These results support hypothesis 3.

### 3.2 Semantic integration on the sentence level

#### 3.2.1 Fixed effects

Similar to the findings for the retrieval of word meanings and in accordance with our expectations, there was a significant negative fixed effect of time on task, *b* = −0.44 (*SE* = 0.06), *z* = −7.57, *p* < 0.001. Thus, on average, faster responses were also more accurate across tasks and persons. This result supports hypothesis 4. Mean performance per grade is given in Table 2. Performance did not differ significantly between grades 1 and 2, *b* = −0.07 (*SE* = 0.23), *z* = −0.33, *p* > 0.05, and 2 and 3, *b* = 0.19 (*SE* = 0.17), *z* = 1.08, *p* > 0.05, while it differed between grades 3 and 4, *b* = 0.54 (*SE* = 0.22), *z* = 2.48, *p* < 0.05.

#### 3.2.2 Random effects

**Figure 3:**
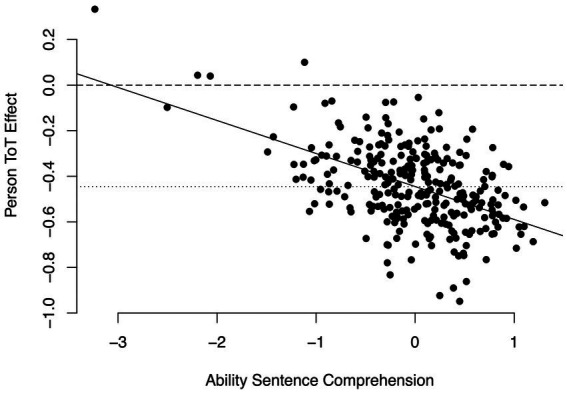
Person-specific time on task-effects in the sentence verification task. Continuous line: Linear regression of person-specific time on task effects on ability. Dashed line: Time on task effect of zero. Dotted line: fixed time on task effect.

*Persons*. First, we examined whether there was variance in time on task effects across persons. We employed the same procedure as with the retrieval of word meanings, comparing the fit of a model M1p_sentence_ that specified a random effect of time on task across persons, but no covariance between random slope and intercept, to the fit of a model M0p_sentence_ that constrained this variance component to be zero and only specified a random person intercept. This comparison indicated that model M1p_sentence_ fit the data significantly better, *χ*^2^(1) = 23.14, *p* < 0.001. This meant that persons differed in how steep the time on task slope was. In a next step, we examined if this variance would be explained by persons’ skills. We estimated a model M2_sentence_ that, in addition to the random slope of time on task across persons, estimated the variance between the random time on task slope across persons and the random person intercept. This model indicated that random person intercept and time on task slope were negatively correlated at *r* = −0.38. As with the retrieval of word meanings, we tested this correlation for significance by comparing the fit of models M2_sentence_ and M1p_sentence_. This comparison indicated that M2_sentence_ fit the data significantly better, *χ*^2^(1) = 7.65, *p* < 0.01. Combining the fixed time on task effect and a posteriori estimates of the random person specific deviations from the fixed time on task effect revealed that person-specific time on task effects were consistently negative with only 4 exceptions, and increasingly so with increasing skill (see Figure 3). This meant that especially for skilled sentence comprehenders, accurate responses were also fast. These results support hypothesis 5.

**Figure 4:**
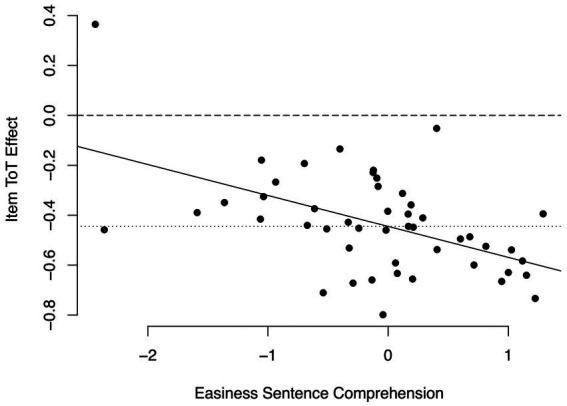
Item-specific time on task-effects in the sentence verification task. Continuous line: Linear regression of item-specific time on task effects on item easiness. Dashed line: Time on task effect of zero. Dotted line: fixed time on task effect.

*Items*. As in the previous analyses, we first examined whether time on task slopes varied across items. We compared a model M1i_sentence_ that specified a random time on task slope across items, but constrained the covariance between the random item slope and intercept to zero, to a model M0i_sentence_ that constrained the random time on task slope across items to be zero. This comparison indicated that model M1i_sentence_ fit the data significantly better than model M0i_sentence_, *χ*^2^(1) = 23.95, *p* < 0.001. Model M2_sentence_ specified the covariance between the random time on task slope across items and the random intercept as an additional parameter and revealed a negative correlation between the random slope and intercept of *r* = −0.42. Comparing this model’s fit to the fit of model M1i_sentence_ revealed that model M2_sentence_ fit the data better than model M1i_sentence_, χ^2^(1) = 4.64, *p* < 0.05. Combining the fixed time on task effect and the a posteriori estimates of the item-specific random time on task effects revealed that item-specific time on task effects were negative with one exception, but it was especially in easy items that fast responses were associated with correct responses (see Figure 4). In (relatively) difficult items, the item-specific time on task effects were negative as well, but to a smaller extent. These results replicated closely the pattern we found for the retrieval of word meanings, and support hypothesis 6.

## 4 Discussion

The present research investigated whether listening component tasks on the word (semantic categorization) and sentence levels (sentence verification) capture cognitive efficiency patterns that are characteristic of automatized processing. Specifically, we examined time on task effects as an indicator of whether response times systematically predict accuracy, reflecting the efficiency of semantic retrieval and integration processes during listening. In accordance with earlier research on automatized skills, we expected the tasks to show that quicker responses are associated with higher accuracy, and that this effect would be more pronounced for stronger comprehenders and easier items. The results confirmed the expected patterns, as quicker responses went along with higher accuracy for both the retrieval of word meanings and sentence-level semantic integration, thus indicating that the tasks captured meaningful variance in the efficiency of these processes.

As for the differences between individuals, person-level random intercepts and slopes were negatively correlated, implying that responding quickly was especially important for the accuracy of more skilled comprehenders. Again, this was the case for both word- and sentence level semantic processes. This result is consistent with the notion that the more routinized these skills become, the more they rely on quick and automatic processing. Although this was also the case for less accurate individuals in both semantic processes, their correlations were weaker in both tasks. For items, the negative correlation between the item-specific intercept and slope showed that quicker responses were more strongly correlated with higher accuracy when items had a higher mean accuracy, i.e., were easier to answer. Thus, especially for easy items, automatic processing was important for accurate responses. Again, this was the case for the semantic categorization task and the sentence verification task.

These results are in line with the idea that oral language comprehension on the word and sentence levels relies on automatized access to meaning rather than slow, deliberate processing. The observed time on task effects suggest that the two listening tasks successfully modeled this efficiency. Especially when comprehenders are more skilled or when the language input is easy to comprehend, it is important that language information is processed automatically with little cognitive effort. When word or sentence meanings were not retrieved correctly, i.e., when children did not understand the meaning of the words or sentences, they required more time to process the input before responding. This could result from a semantic search requiring a more deliberate cognitive effort and a higher strain on working memory, in line with the cognitive model of listening (Imhof, 2010) or the verbal efficiency theory (Perfetti, 1985). Nevertheless, this process took longer than the responses to correctly answered items in both tasks, even for children with lower accuracy, indicating that no matter the skill level of the children, access to the correct word or sentence meanings did not require as much cognitive effort. Therefore, in the present study with elementary school children, we could not detect different processing types as proposed for reading skills by the compensatory-encoding model (Walczyk, 2000), but listening skills at this age may have already been too advanced and the language input too easy to find these differences.

These results are nevertheless in line with studies on reading processes, where automatization on different levels has been shown to be an important predictor of successful comprehension (Roembke et al., 2019; Schindler and Richter, 2018). It can be argued that sentence- or text-level processes are not entirely automatic as these rely on memory to integrate prior knowledge or draw inferences, but the automaticity of lexical access and propositional encoding decrease the reliance on deliberate cognitive processing (Hui and Godfroid, 2021; Perfetti, 1985). Semantic integration in sentences relies on both of these processes, which in turn may mediate its efficiency.

Regarding differences between grade levels, a significant difference in accuracy was found between Grades 3 and 4, indicating a modest increase in listening efficiency toward the end of elementary school. However, no significant incremental gains were observed between the earlier grades. This contrasts with previous findings on similar processes in reading and listening, where larger developmental improvements in accuracy were found across the early school years (e.g., Dahdah et al., 2025; Richter et al., 2012; Verhoeven and Van Leeuwe, 2008). The smaller early gains in the present data may reflect cohort differences. Moreover, because the data are cross-sectional, developmental inferences should be made with caution.

In terms of the limitations of the present study, the assessments were limited to children in grades one through four. As listening comprehension – at least in one’s first language – begins to develop considerably earlier than elementary school (e.g., Fernald et al., 2006), semantic comprehension processes on the word and sentence levels may have already reached a point where they are predominantly automatized. It is reasonable to assume that semantic retrieval prior to elementary school may not yet occur automatically and require more cognitive effort, similar to reading processes (Walczyk, 2000; Walczyk et al., 2007). A look at these processes at earlier ages could thus give insight into when these processes switch from deliberate to automatic execution. Furthermore, the present analyses were based on two samples of children who each solved either the semantic categorization or the sentence verification task. Therefore, it was not possible to assess the correlations between response times and accuracy between both tasks. Similarly, it was not possible to directly compare the time on task effects between the word and sentence levels. Furthermore, we did not include additional measures such as intelligence or broader language abilities. Collecting these data was not feasible within the limited testing time available in the classroom setting. However, by including class and grade level into the analyses, we were able to at least in part account for variance related to these variables. Moreover, the items in the study were recorded in a studio environment and articulated clearly at a speech rate that was easy to follow for elementary school children. Whereas this was appropriate for examining whether our tasks capture efficiency under controlled conditions, it remains unclear how well the same efficiency indicators would perform in more realistic listening environments. Future studies could therefore incorporate more varied and challenging listening environments that include varying speech rates, less clear articulation, noise, or other signal degrading characteristics. This higher difficulty could put a higher burden on attentional resources (Murgia, 2024; Wild et al., 2012) but may also lead to a higher reliance on the automaticity of comprehension processes. Examining task performance under such conditions would help determine the generalizability of efficiency-based listening assessments.

In sum, the results of this study show that for auditory semantic processes on the word and sentence levels, shorter response times were systematically associated with higher accuracy, particularly among skilled comprehenders and for easier items. This pattern supports the assumption that our tasks capture cognitive efficiency in line with automatized processing, rather than reflecting deliberate or effortful processing.

The present results also have important implications for the assessment of listening component skills. They suggest that response latencies can provide valid information about the efficiency of semantic listening processes and can therefore complement accuracy-based measures in the evaluation of language proficiency. In other words, interpreting shorter response times as reflecting cognitive efficiency appears reasonable for these types of listening tasks. Future research should extend this approach to more complex listening skill components, such as coherence building or inference generation, and to younger children for which these processes are not yet automatized, in order to determine the advantages and limitations of efficiency-based assessments in listening comprehension.

## Data Availability

The datasets presented in this study and the analysis script needed to reproduce the reported analyses are openly available in an OSF repository at: https://osf.io/gcm3n.
